# A cohort study of *Chlamydia trachomatis* treatment failure in women: a study protocol

**DOI:** 10.1186/1471-2334-13-379

**Published:** 2013-08-17

**Authors:** Jane S Hocking, Lenka A Vodstrcil, Wilhelmina M Huston, Peter Timms, Marcus Y Chen, Karen Worthington, Ruthy McIver, Sepehr N Tabrizi

**Affiliations:** 1 Melbourne School of Population and Global Health, University of Melbourne, Level 3, 207 Bouverie St, Carlton 3053, Victoria, Australia; 2 Murdoch Children’s Research Institute, Parkville 3052, Victoria, Austrlaia; 3 Institute of Health and Biomedical Innovation, Queensland University of Technology, 60 Musk Ave, Kelvin Grove, Brisbane 4057, Queensland, Australia; 4 Melbourne Sexual Health Centre, 580 Swanston Street, Carlton 3053, Victoria, Australia; 5 Sydney Sexual Health Centre, Sydney Hospital, Macquarie Street, Sydney 2001, New South Wales, Australia; 6 Department of Microbiology and Infectious Diseases, the Royal Women’s Hospital, Parkville 3052, Victorian, Australia; 7 Department of Obstetrics and Gynaecology, University of Melbourne, Carlton 3053, Victoria, Australia

**Keywords:** *Chlamydia trachomatis*, Azithromycin, Treatment failure, Re-infection, Sexually transmitted infections

## Abstract

**Background:**

*Chlamydia trachomatis* is the most commonly diagnosed bacterial sexually transmitted infection in the developed world and diagnosis rates have increased dramatically over the last decade. Repeat infections of chlamydia are very common and may represent re-infection from an untreated partner or treatment failure. The aim of this cohort study is to estimate the proportion of women infected with chlamydia who experience treatment failure after treatment with 1 gram azithromycin.

**Methods/design:**

This cohort study will follow women diagnosed with chlamydia for up to 56 days post treatment. Women will provide weekly genital specimens for further assay. The primary outcome is the proportion of women who are classified as having treatment failure 28, 42 or 56 days after recruitment. Comprehensive sexual behavior data collection and the detection of Y chromosome DNA and high discriminatory chlamydial genotyping will be used to differentiate between chlamydia re-infection and treatment failure. Azithromycin levels in high-vaginal specimens will be measured using a validated liquid chromatography – tandem mass spectrometry method to assess whether poor azithromycin absorption could be a cause of treatment failure. Chlamydia culture and minimal inhibitory concentrations will be performed to further characterize the chlamydia infections.

**Discussion:**

Distinguishing between treatment failure and re-infection is important in order to refine treatment recommendations and focus infection control mechanisms. If a large proportion of repeat chlamydia infections are due to antibiotic treatment failure, then international recommendations on chlamydia treatment may need to be re-evaluated. If most are re-infections, then strategies to expedite partner treatment are necessary.

## Background

Over 100 million men and women worldwide are infected with chlamydia at any point in time [1]. It is the most commonly reported bacterial sexually transmitted infection (STI) in developed countries, with over 1.4 million cases reported in the United States in 2011 [2]. Left untreated, chlamydia can ascend from the endocervix to the upper genital tract in women and cause pelvic inflammatory disease (PID) which can increase the risk of developing fallopian tube scarring, potentially leading to ectopic pregnancy, tubal infertility and chronic pelvic pain [3-5]. In addition, genital infection in pregnant women increases the risk of preterm delivery, can be passed on to the baby during vaginal delivery and may result in eye and lung infections in the new born [3,6]. Infection with genital chlamydia can also increase the risk of HIV acquisition in both men and women, and cause epididymo-orchitis in men [4,7].

Repeat chlamydia infections are common following treatment. Among women who tested positive in an Australian cohort of 1116 young women who were treated at recruitment, 18% tested positive again at 3 months (95% CI: 8%, 34%) [8]. In the UK, a prospective cohort of 16 to 24 year old women treated for chlamydia in general practice, reported a repeat infection rate of 29.9% per year (95% CI: 19.7%, 45.4%) [9]. Another cohort of adolescent women in the US reported a repeat infection rate of 34% per year [10] and a recent systematic review of chlamydia repeat infection following treatment found that the overall median proportion testing positive again for chlamydia was 13.9% [11].

Repeat infections are generally considered to be re-infections through exposure to an infected partner. However, emerging evidence suggests that treatment failure following azithromycin may account for a substantial proportion and this has led to considerable debate in the medical and scientific literature [12-16]. Among female participants in a partner treatment trial who reported no sexual intercourse after treatment, 22 of 289 (8%; 95% CI: 5%, 11%) had persistent infection suggestive of treatment failure at follow up [17]. Similarly, the treatment failure rate in a cohort of adolescent females was 7.9% (95% CI: 4%, 10.1%) [10]. Both of these studies attempted to differentiate between re-infection and treatment failure using sexual behavior questionnaires. Batteiger et al. (2010) included genotyping as a further tool to identify re-infections, but these studies were based on self-report of sexual behavior which may have been unreliable.

The recommended first line treatment for uncomplicated genital chlamydia infection in most developed countries is a 1 gram dose of the macrolide antibiotic, azithromycin [18-20]. Although doxycycline 100 mg twice daily for 7 days is a second line treatment for uncomplicated chlamydia, it is not widely used because there are concerns about compliance given the longer duration of treatment [21]. A meta-analysis of chlamydia treatment reported a 97% cure rate for azithromycin and 98% for doxycycline [22]. Of the 12 trials included in the meta-analysis, 11 used culture or immunoassay rather than the more sensitive nucleic acid amplification tests (NAAT) to determine microbial cure at study end [22]. Given the use of culture rather than NAATs, it is possible that the treatment efficacies in these trials were over-estimated [12,13,23].

Governments throughout the developed world are pushing for increased chlamydia testing and the Australian government has invested in a large chlamydia screening randomized controlled trial [24]. It is imperative, therefore, to rigorously investigate the adequacy of currently recommended treatment for chlamydia. We describe here a cohort study, the Australian Chlamydia Treatment Study (ACTS), that aims to measure the proportion of chlamydia infected women who fail treatment when treated with 1 gram azithromycin using advanced microbiological techniques to differentiate between re-infection and treatment failure.

### Research aim

The primary aim is to estimate the proportion of women infected with chlamydia who fail treatment with 1 gram azithromycin, the most widely recommended first line treatment for genital chlamydia infection [18-20]. The secondary aim is to determine the role of *C. trachomatis* organism load, azithromycin tissue absorption of azithromycin and antimicrobial resistance in chlamydia treatment failure.

## Methods/design

### Study design and setting

This is a cohort study of women who test positive for genital chlamydia at one of two large, publically funded sexual health centres in Melbourne and Sydney, Australia. Participants will be followed up for up to 56 days post treatment and will provide weekly genital specimens for further assay.

### Duration of study

Recruitment commenced in October of 2012 and is expected to continue until October 2014, with results reported late 2015.

### Participant eligibility

Women who are diagnosed with genital chlamydia and who meet eligibility criteria will be invited to participate in the study. The study is limited to women because of their increased risk of serious chlamydia related complications.

The inclusion criteria are:

• Female;

• Positive test for genital chlamydia using Nucleic Acid Amplification Test polymerase chain reaction [PCR] (performed on specimens collected from women recruited from Sydney Sexual Health Centre) or strand-displacement assay (performed on specimens collection from women recruited from Melbourne Sexual Health Centre);

• Age ≥16 years;

• Adequate English and comprehension skills to give informed consent;

• Able to attend their recruitment clinic for follow up and specimen collection at day 7;

• Resides in a jurisdiction serviced by one of the two clinics and plan to stay in the jurisdiction for the next 8 weeks.

The exclusion criteria are:

• Current infection detected as a part of a routine test for re-infection [18];

• Concomitant infection with another bacterial STI;

• Concurrent PID;

• Self report of antibiotic use in the last 2 weeks;

• Current commercial sex work;

• Women who do not wish to receive study packs by post;

• Women who do not have a mobile phone;

• HIV positive status;

• Concurrent medication likely to significantly interact with azithromycin (e.g. cyclosporine, digoxin);

• Known macrolide allergy.

### Recruitment

Women who have tested positive for chlamydia will be seen by a research nurse at a participating clinic at point of treatment. The research nurse will explain the study to them, assess eligibility and take informed consent. The research nurse will collect specimens for further testing (Table 1) and provide treatment with a single dose of 1 gram azithromycin. The nurse will observe the participant as she takes her treatment to ensure compliance. This is classified as the baseline visit (Day 0). All clinic visits, pathology costs and treatment for participants and their partners will be provided free of charge.

**Table 1:** Specimen collection by study timeline.

**Specimen collection and testing**										**If PCR positive @ d28, 42 or 56**
Day	0^d^	7	14	21	28	35	42	49	56	
Number of swabs collected	4	2	2	2	2	2	2	2	2	4-5
Culture	X									X
PCR^a^	X	X	X	X		X		X		X
Chlamydia organism load^a^	X	X	X	X	X	X	X	X	X	X
Genotype^a^	X	X	X	X	X	X	X	X	X	X
Sequencing^a^	X	X	X	X	X	X	X	X	X	X
Y-chromosome^a^		X	X	X	X	X	X	X	X	X
β-globin^a^	X	X	X	X	X	X	X	X	X	X
Test of cure^b^					X		X		X	
Az absorption^a^		X								
Blood	X									X
Rectal swab^c^										X
Location	Clinic^e^	Clinic^e^	Home^f^	Home^f^	Home^f^	Home^f^	Home^f^	Home^f^	Home^f^	Clinic^e^

^a^Processed in batches throughout the course of the study.

^b^Chlamydia PCR done in real time to report results back to participants and treat any women diagnosed chlamydia positive.

^c^If anal sex reported.

^d^Day 0 = baseline or at time of recruitment.

^e^Swabs collected by research nurse and put immediately into the appropriate medium.

^f^Self-collected high-vaginal swabs.

Key: X = specimen processed for this experiment, PCR polymerase chain reaction, Az *absorption* azithromycin absorption, d day.

### Primary outcome

**Figure 1:**
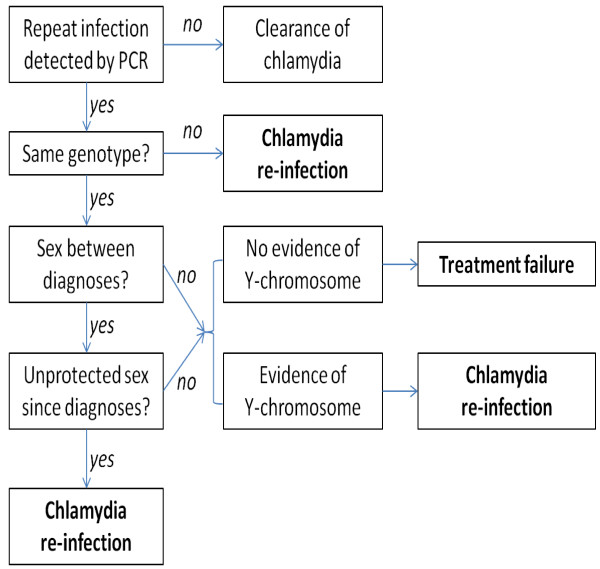
Algorithm for classifying treatment failure.

The primary outcome is the proportion of women who are classified as having chlamydia treatment failure following detection of chlamydia on a self-collected high-vaginal specimen using a polymerase chain reaction (PCR – see below) Specimens will be collected for testing on days 28, 42 or 56 after recruitment (referred to as ‘test of cure’ specimens for the purpose of this study) according to the algorithm in Figure 1. This algorithm follows a previously described method [10] with the addition of the detection of Y chromosome DNA and performing high discriminatory chlamydial genotyping. Y chromosome DNA will be used to validate sexual behaviour data because it can be detected in the vagina for up to 14 days after unprotected intercourse with a male partner [25,26]. Specimens will be collected from participants every 7 days for Y chromosome detection and genotyping will be undertaken on any chlamydia positive specimens collected on days 28, 42 or 56 after recruitment. Chlamydial genotype fingerprinting will be performed to determine any differences in chlamydia strain/serovar between a participant’s baseline specimen and any repeat positive specimens detected at day 28, 42 or 56 [27]. If a woman has the same serovar present at follow up, further testing by multilocus sequence typing (MLST) targeting *ompA* and five house-keeping genes will be undertaken [28,29]. Detection of same MLST type without detection of Y chromosome DNA or any reports of unprotected sex will be classified as treatment failure. If the woman has a different serovar, it will be classified as a re-infection. A repeat positive chlamydia diagnosis will also be classified as a re-infection if the woman’s repeat specimen has the same MLST type and Y chromosome is detected or she reports unprotected sexual contact.

### Study endpoint

**Figure 2:**
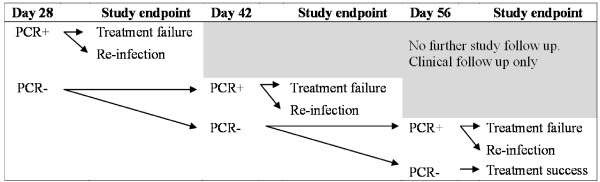
Classification of study endpoint.

A study participant will reach the study endpoint when she is diagnosed with treatment failure, re-infection or treatment success. The study endpoint will be measured by a test of cure PCR conducted after 28, 42 or 56 days follow up according to Figure 2.

### Further classification of treatment failure

Treatment failure will be further classified as:

*Poor azithromycin absorption:* There is no vomiting or diarrhoea reported after treatment but low levels of azithromycin concentration are found in the vaginal cells [30,31] and the MIC for the initial chlamydia culture is within reported range.

*Persistent chlamydia not responding to azithromycin:* Azithromycin absorption shows adequate levels of azithromycin in the vaginal cells, the minimal inhibitory concentration (MIC) for the initial chlamydia culture is within reported range and the subsequent chlamydia culture is negative [13,23,32,33].

*Reduced antimicrobial susceptibility and resistance:* Some viable infectious chlamydia is present when cultured with antibiotic series compared with cultures with no antibiotic series. The MIC for initial chlamydia culture is elevated compared with reported range [13,15,32,34,35]. Evidence of 23S rRNA gene mutation may or may not be present.

### Specimen collection and testing

Participants will be asked to return to the clinic for follow up testing at day 7 so that the research nurse can collect further specimens. Thereafter, weekly high-vaginal specimens will be self-collected at home and mailed in a postage-paid envelope to the study centre. Participants will receive weekly short message service (SMS) prompts reminding them to return specimens and questionnaires and will be telephoned if they test chlamydia positive again throughout the study. These women will be requested to attend the clinic for treatment and further specimen collection. Table 1 outlines the number of specimens collected and the tests to be undertaken at each stage of the study.

### Chlamydia polymerase chain reaction (PCR)

Test of cure swab specimens collected on day 28, 42 and day 56 will be placed in Cobas® PCR Media (Roche Diagnostics) and will be tested in batches on COBAS 4800 CT/NG (Roche Diagnostics). The remaining eluted DNA from COBAS 4800 will be stored at −80°C until required for further assay. For specimens collected at other time points, swabs collected will be rotated for 30 seconds in 1 ml of phosphate buffered saline (PBS) and stored at −80°C until further testing required. Specimens will be subsequently extracted with MagNA Pure 96 (Roche Diagnostics) using 200 μl of the cellular suspension in conjunction with the total nucleic acid isolation kit. Eluted nucleic acid of 100 μl will be tested as required.

### Genotyping & quantification of organism load

Chlamydial fingerprinting will be conducted on specimens that are PCR positive at baseline and at test of cure. We will determine the organism load, identify the chlamydia serovar(s) (genovar) of each infection through a series of quantitative PCR assays to establish whether or not the genotype of the chlamydia detected in those women who have a repeat positive is the same as the type present at baseline [27]. Initial qPCR primers and probes have been designed to predict antigenic differences in major outer membrane protein (MOMP) to determine the serovar as previously described [27,36-38]. The primary chlamydia group-specific multiplex quantitative PCR will target conserved regions of the *ompA* gene specific to all chlamydia serovar groups, including the B group (serovars B, E, D, L1, and L2), C group (serovars A, C, H, I, J, K, and L3) or intermediate group (serovars F and G) serovars. This assay will enable quantification of organism load and will be used to direct serovar-specific PCRs to determine serovars present in the specimen, including possibility of mixed serovars [27,39].

Chlamydia organism load in each specimen will be quantified by comparing the crossing-threshold of each specimen to the crossing-threshold of a standard curve constructed by amplifying different known copy numbers of the *ompA* gene. The beta globin gene, present at one haploid copy per nucleated cells [40], will also be quantified using qPCR to assess specimen adequacy as well as to measure sampling variability between participants and specimens by correlation with the number of eukaryotic cells collected. The quantity of chlamydia will be divided by the number of eukaryotic cells and expressed as the number of organisms present per 100 eukaryotic cells [40].

Specimens from participants where the same serovar has been detected at baseline and follow up will undergo further discriminatory confirmation of relatedness using sequencing of *ompA* gene, as well as five house-keeping genes (*hctB,* CT058, CT144, CT172, and CT682 [*pbpB*]) utilizing a multilocus sequence typing (MLST) approach [28,29,41,42]. This will include the amplification and sequencing [29,43,44] followed by determining MLST type using *C. trachomatis* MLST database (mlstdb.bmc.uu.se).

### DNA sequencing to identify mutation in 23S rRNA gene

Each specimen will be subjected to DNA sequencing (as described above) of PCR-amplified region of the 23S rRNA gene flanking the positions 2057 and 2611. Presence of changes in three locations, A2057G, A2059G and T2611C (*E. coli* numbering), will be determined as they are associated with increased macrolide resistance [45]. The data arising from this experiment will be correlated with MICs as described below.

### Detection of Y chromosome

A separate swab will be collected each week and placed in 400 μl of PBS for Y-chromosome detection as evidence of unprotected sexual exposure. Real-time PCR directed at amplification of a region of the Y chromosome targeting SRY (sex determining region Y) will be conducted [25,26].

### Culture

Endocervical specimens collected by speculum examination will be used for chlamydia culture. The research nurse will place the swab immediately into culture medium, store at −80°C, and courier to the laboratory on dry ice for processing. Chlamydia isolates will be cultured on HEp-2 to propagate each isolate prior to progressing to MICs. Specimens will be prepared by vortexing before addition to 20 hour HEp-2 cultures in antibiotic free DMEM supplemented with 10% serum. Cultures will be incubated at 37°C, 5% CO_2_ for 48 hours and viable infectious chlamydia elementary bodies will be harvested by mild sonication. A serial dilution of the specimen cultured onto coverslips in 24 well plates with 20 hr growth 2 × 10^5^ HEp-2 cells to determine the viable infectious yield [46].

### Minimum inhibitory concentration (MIC)

MIC will be conducted by culturing chlamydia on 20 hr HEp-2 cells. HEp-2 cells will be used for all MIC determinations as they are the most relevant immortalised cell line (cervical), are widely used, and have previously been shown to have consistent performance in MIC assays when compared to other cell lines [35]. Low passage clinical isolates prepared above will be inoculated onto the HEp-2 cells at 2500 and 5000 IFU per well. Cultures will be centrifuged for 1 hr (1200 × g) to ensure equal infection rates. These inoculums will be used as they have previously been reported to be an appropriate dose to predict the MCC (minimum chlamydicidal concentration) and are adequate numbers to accurately assess the inclusions formed [35]. The antibiotic will be titrated into the infected cultures at 4 hours after addition of the chlamydia using a twofold dilution series from 140 g/ml to 0.008 g/ml (in fresh media with cycloheximide 1 g/ml). Antibiotic will be freshly prepared as per the manufacturer’s instructions for each experiment. The numbers of correctly formed inclusions will be determined by methanol fixing the coverslips at 30 hours and stained to allow clear visualisation and counting of the inclusions on the microscope (Chlamydia Cel, Vital diagnostics). The MIC will be defined as the concentration at which no typical inclusions can be seen on the entire coverslip by microscopy examination.

### Azithromycin absorption

High-vaginal specimens collected by the research nurse at the day 7 visit will be preserved in 1 ml of 100% methanol and stored at −80°C prior to analysis. Azithromycin levels are thought to remain well above the reported MIC for chlamydia for between 10 to 14 days post-treatment with 1 g dose [30,31]. Azithromycin levels in high-vaginal material (cells and mucus) will be measured using a validated liquid chromatography – tandem mass spectrometry method (LC-MS/MS) [47-50]. An azithromycin standard curve will be prepared in high-vaginal specimens from a separate individual not exposed to azithromycin, and azithromycin concentrations detected by LC-MS/MS will be normalised to lipid concentrations [50]. Azithromycin concentrations in high-vaginal specimens will be normalised to membrane lipid concentrations and then calculated using the standard curve.

### Immunological markers

Blood specimens will be taken at the recruitment visit and from all women who return a positive test of cure test. Serum will be analyzed for chlamydia antibodies titres against peptide and protein antigens using previously described methods [51,52].

### Chlamydia test results and management

Women will be treated with 1 gram azithromycin according to site protocol at the time of recruitment. Those who have a positive test of cure by PCR at day 28, 42 or 56 will be asked to return to the clinic for further treatment of doxycycline 100 mg twice daily for ten days. All laboratory assay results will be forwarded to the study centre and entered into the study database.

### Partner notification

At the time of recruitment, the research nurse will explain the importance of treating sexual partners and avoiding sexual contact for 7 days. Multiple strategies to support partner treatment will be explored. Participants will be given a business card that contains the web address for *Let Them Know* (http://www.letthemknow.org.au/), a partner notification service for sexual contacts of chlamydia and other STIs. *Let them know* allows individuals to send a message to a partner, either anonymously or named, by SMS or email. If preferred, women may direct their partners to call the research nurse for information and a referral to appropriate clinical care. Women may also consent to have their sexual partner(s) contacted for a telephone consultation and, if appropriate, prescribed azithromycin treatment for partner or mail delivery. This method has been used in several studies at Melbourne Sexual Health Centre and has been very effective at maximizing partner treatment [53,54].

### Data collection

Participants will be asked to complete questionnaires at the time of recruitment and each week until they reach study endpoint. The following data will be collected:

• Age, height, weight

• Genital symptoms

• Reason for attending clinic for their initial test

• Current medications including contraceptives and concurrent antibiotics

• Date of last menses

• Sexual practice data including number of sexual partners, type of sexual partners (casual, occasional, once off or regular), type of sexual contact (vaginal or anal sex), condom use; and

• Whether partners have been notified and treated

### Data analysis

#### Sample size estimates

To detect a treatment failure risk of 8% with a 95% CI of 5.5%, 10.5%, 450 women are required. If the risk of treatment failure is observed to be 3% (as reported by the earlier meta-analysis [22]), then a sample size of 450 generates a 95% CI of (1.7%, 5.0%). To account for a loss to follow up of 15% based on previous studies [8], 520 women will be recruited.

### Statistical analysis

The population risk of treatment failure will be estimated as the observed proportion of women in the sample with treatment failure, with 95% CI calculated assuming an underlying binomial distribution. Multinomial logistic regression will be used to explore the association between the three-level outcome variable (treatment failure, treatment success, re-infection) and risk factors such as the impact of organism load, azithromycin absorption and MIC. One advantage of a multinominal model over two separate logistic regression models (one comparing the prevalence of treatment failure with treatment success, the other comparing the prevalence of re-infection with treatment success) is that the question of whether treatment failure and re-infection share common risk factors can be addressed formally by comparing estimated odds ratios generated by the same model. Linear regression analyses will investigate trends in chlamydia organism load over time and explore any associations with symptoms, chlamydia serovar and participant age. Both linear and multinomial regression models will be implemented with fixed and random regression effects to accommodate the longitudinal and repeated measures nature of the data. All analyses will be conducted in Stata 12.0.

### Loss to follow up

The provision of reimbursement payments throughout the study is paramount to maximize retention. Women will be reimbursed with vouchers totalling up to $100 to cover their transport and time costs during the study. This will be broken down into smaller payments, $25 at their first follow up visit, $10 for their day 14 specimen, $10 for their day 21 specimen, $25 for their day 28 specimen and then $30 upon completion of the study (day 56 specimen or earlier if repeat positive). Reimbursements will reflect how many specimens and questionnaires are returned.

## Discussion

Distinguishing between chlamydia re-infection and treatment failure is important to focus treatment recommendations and infection control mechanisms. For example, if most repeat infections in this study are found to be re-infections, then strategies to expedite partner treatment are necessary. If many repeat infections are due to antibiotic treatment failure, then international recommendations on chlamydia treatment need to be re-evaluated.

Previous studies that have reported an azithromycin treatment failure rate of 2-3% utilized test of cure by chlamydia culture which, in the last decade, has been replaced by more sensitive PCR testing [22]. Chlamydia treatment studies that have utilized PCR testing have reported much higher treatment failure rates of up to 8%; however these studies were not designed to reliably distinguish between re-infections and treatment failure [10,17]. Our study is one of the first to use both robust molecular microbiological testing and validated behavioural data so that treatment failure rates can be more accurately estimated. We hypothesize that the treatment failure rate will be closer to 8%.

If our hypothesis is correct and 8%, rather than 2-3%, fail chlamydia treatment with azithromycin, then nearly 3,000 women in Australia and 70,000 women in the USA were inadequately treated for chlamydia in 2011 [2,55]. Treatment failure will lead to persistent infection with a longer duration of infection and increased risk of complications as well as continued transmission in the population. Further, as chlamydia culture has been rarely undertaken over the last decade, there is little monitoring of chlamydia antibiotic susceptibility, and the role of antimicrobial resistance in treatment failure is unknown [35]. The ability to differentiate between re-infection and treatment failure is a strength of this study. Techniques used will include measuring azithromycin absorption in genital cells, chlamydia culture, genotyping, DNA sequencing and antimicrobial sensitivity determinations of the chlamydia isolates obtained. The results from this study will inform discussions about chlamydia treatment failure and help to establish whether 1 gram azithromycin, the most widely recommended chlamydia treatment internationally, is appropriate. If treatment failure is confirmed to be higher than previously estimated, then further chlamydia treatment trials to suggest changes to international treatment guidelines will be indicated.

### Ethics approval

Ethical approval for this study was granted by the Alfred Hospital Ethics Committee and the Southern Eastern Sydney Local Health District Human Research Ethics Committee (Southern Sector).

## Competing interests

The authors have no competing interests to declare.

## Authors’ contributions

All authors designed and co-authored the protocol. JSH, WMH, PT, MC and ST obtained the grant funding to conduct the study. LV, KW and RM have developed the recruitment methodology. All authors read and approved the final manuscript.

## Pre-publication history

The pre-publication history for this paper can be accessed here:

http://www.biomedcentral.com/1471-2334/13/379/prepub
